# Discussion

**Published:** 1918-12

**Authors:** 


### Discussion.

MAJOR CARLSON said that in view of the prominence given to serum procedure in the prevention of tetanus, he should like to ask if there was any evidence at all in the data collected during the war as to the prophylactic value of serum. What right have we to ascribe the decrease in tetanus to the use of serum rather than to the careful use of the knife?

MAJOR-GENERAL SIR DAVID BRUCE replied that he thought there was no doubt that the fall in the number of cases must be due to prophylactic injection. The excision and thorough surgical treatment of wounds had only come on within the last year so it could have nothing to do with the incidence of tetanus at the beginning of the war.

Major Gibson said that in regard to the effect of surgery he should like to say a few words. By the "effect of surgery" he said he meant the early operative treatment of wounds. The early incision of wounds began about the middle of 1917, and beyond the very remarkable drop in the incidence of tetanus, due to prophylactic injection, the practice of general excision had a further effect on the incidence of the disease. From 103 per 100,000 in pre-incision days, the rate of tetanus had fallen to 24 per 100,000 in February, 1918. In May there were only 12 cases per 100,000 and 8 per 100,000 in June.

Colonel Cummins said that he desired to congratulate the members of the American Medical Research Society on their good fortune in being able to arrange for a paper on Tetanus by Sir David Bruce, who, as Chairman of the Tetanus Committee at the War Office, has had a unique opportunity of investigating this subject.

While thoroughly concurring with what Sir David Bruce had said, the speaker remarked that there were still one or two points on which the experience of those in France tended to differ from that of the workers in England.

In the hospitals of the British Expeditionary Force the tetanus cases arose among wounded who had just arrived from the "line", and it was therefore natural that the incubation period should be, on the average, shorter than that recorded in England. The fact that very little hospital accommodation existed in France in 1914 led to the rapid evacuation of severe cases to England, thus probably helping to explain why the incubation period noted by Sir David Bruce at the beginning of the war was so much shorter than in the past two years. The greater the facilities for retaining severely wounded cases in France, the smaller would be the number of early cases of tetanus developing in England.

Commenting on the statement of Sir David Bruce that the majority of the cases of tetanus arising in England in 1914 were men who had not received a prophylactic inoculation, Colonel Cummins reminded his audience that this was a tribute to the value of prophylactic inoculation, rather than a proof that none had been given at that time. As a matter of fact, although supplies of serum were not on a scale to enable universal inoculation to be practised, still the severely wounded cases nearly always received it.

During the battle of the Aisne, when the change of bases from the northern ports to St. Nazaire interrupted the forwarding of medical supplies, we were still able to obtain anti-tetanic serum through the kindness of our French Allies, and with the assistance of our American friends. Mr. Robert Bacon, at that time working with the American Red Cross, placed his cars, his time, and his initiative, completely at our disposal, and was instrumental in bringing up supplies of anti-tetanic serum, kindly placed at our disposal by the Pharmacie Centrale of the French Army and the Pasteur Institute. In this way many lives and much suffering must have been saved.

